# A scoping review of gestational diabetes mellitus healthcare: experiences of care reported by pregnant women internationally

**DOI:** 10.1186/s12884-022-04931-5

**Published:** 2022-08-08

**Authors:** Sheila Pham, Kate Churruca, Louise A. Ellis, Jeffrey Braithwaite

**Affiliations:** Centre for Healthcare Resilience and Implementation Science, Australian Institute of Health Innovation, Macquarie University, 75 Talavera Rd, North Ryde, NSW 2113 Sydney, Australia

**Keywords:** Gestational diabetes mellitus, Pregnancy, Patient experience, Delivery of healthcare, Scoping review

## Abstract

**Background:**

Gestational diabetes mellitus (GDM) is a condition associated with pregnancy that engenders additional healthcare demand. A growing body of research includes empirical studies focused on pregnant women’s GDM healthcare experiences. The aim of this scoping review is to map findings, highlight gaps and investigate the way research has been conducted into the healthcare experiences of women with GDM.

**Methods:**

A systematic search of primary research using a number of databases was conducted in September 2021. Studies were included if they had an explicit aim of focusing on GDM and included direct reporting of participants’ experiences of healthcare. Key data from each study was extracted into a purposely-designed form and synthesised using descriptive statistics and thematic analysis.

**Results:**

Fifty-seven articles were included in the analysis. The majority of studies used qualitative methodology, and did not have an explicit theoretical orientation. Most studies were conducted in urban areas of high-income countries and recruitment and research was almost fully conducted in clinical and other healthcare settings. Women found inadequate information a key challenge, and support from healthcare providers a critical factor. Experiences of prescribed diet, medication and monitoring greatly varied across settings. Additional costs associated with managing GDM was cited as a problem in some studies. Overall, women reported significant mental distress in relation to their experience of GDM.

**Conclusions:**

This scoping review draws together reported healthcare experiences of pregnant women with GDM from around the world. Commonalities and differences in the global patient experience of GDM healthcare are identified.

**Supplementary Information:**

The online version contains supplementary material available at 10.1186/s12884-022-04931-5.

## Background

Gestational diabetes mellitus (GDM) is defined as any degree of hyperglycaemia recognised for the first time during pregnancy, including type 2 diabetes mellitus diagnosed during pregnancy as well as true GDM which develops in pregnancy [1]. GDM is associated with a number of adverse maternal and neonatal outcomes, including increased birth weight and increased cord-blood serum C-peptide levels [2], as well as greater risk of future diabetes [3].

The global incidence and health burden of GDM is increasing [4] and the cost of healthcare relating to GDM significant. In 2019, the International Diabetes Federation estimated the annual global diabetes-related health expenditure, which includes GDM, reached USD$760 billion [4]. In China, for example, the annual societal economic burden of GDM is estimated to be ¥19.36 billion ($5.59 billion USD) [5].

GDM is estimated to affect 7–10% of all pregnancies worldwide, though the absence of a universal gold standard for screening means it is difficult to achieve an accurate estimation of prevalence [6], and the prevalence of GDM varies considerably depending on the data source used [7]. In Australia, for example, between 2000 and 01 and 2017-18, the rate of diagnosis for GDM tripled from 5.2 to 16.1% (3); furthermore, in 2017-18, there were around 53,700 hospitalisations for a birth event where gestational diabetes was recorded as the principal and/or additional diagnosis [8]. Important risk factors for GDM include being overweight/obese, advanced maternal age and having a family history of diabetes mellitus (DM), with all these risk factors dependent on foreign-born racial/ethnic minority status [9]. However, primarily directing research to understanding risk factors does not necessarily lead to better pregnancy care, particularly where diabetes is concerned, and developing better interventions requires consideration of women’s beliefs, behaviours and social environments [10].

 To date there have been numerous systematic and scoping reviews focused on women’s experiences of GDM, which provide a comprehensive overview of numerous issues. However, gaps remain. In 2014, Nielsen et al. [11] reviewed qualitative and quantitative studies to investigate determinants and barriers to women’s use of GDM healthcare services, finding that although most women expressed commitment to following health professional advice to manage GDM, compliance with treatment was challenging. Their review also noted that only four out of the 58 included studies were conducted in low-income countries. In their follow-up review, Nielsen et al. specifically focused on research from low and middle income countries (LMIC) to examine barriers and facilitators for implementing programs and services for hyperglycaemia in pregnancy in those settings [12] and identified a range of factors such as women reporting treatment is “expensive, troublesome and difficult to follow”.

In 2014, Costi et al. [13] reviewed 22 qualitative studies on women’s experiences of diabetes and diabetes management in pregnancy, including both pre-existing diabetes and GDM. From their synthesis of study findings, they concluded that health professionals need to take a more whole-person approach when treating women with GDM, and that prescribed regimes need to be more accommodating [13]. Another 2014 review by Parsons et al. [14] conducted a narrative meta-synthesis of qualitative studies. Their 16 included studies focused on the experiences of women with GDM, including healthcare support and information, but the focus of their meta-synthesis was focused on perceptions of diabetes risk and views on future diabetes prevention.

In a systematic review of qualitative and survey studies from 2015, Van Ryswyck et al. [15] included 42 studies and had similar findings to Parsons et al. [14], also emphasising their findings regarding the emotional responses of women who have experienced GDM. Specifically, Van Ryswyck et al. [15] identified that women’s experiences ran the gamut of emotions from “very positive to difficult and confusing”, with a clear preference for non-judgmental and positively focused care. Most recently, the 2020 systematic review of qualitative studies by He et al. [16] synthesised findings from 10 studies to argue that understanding the experiences of women with GDM can aid health care professionals to better understand those under their care and to develop more feasible interventions to reduce the risk of DM. A further systematic review of qualitative studies by Craig et al. [17] focused on women’s psychosocial experiences of GDM diagnosis, one important aspect of healthcare experience, highlighting future directions for research into the psychosocial benefits and harms of a GDM diagnosis.

There has been insufficient consideration of epistemological assumptions and other aspects of research design which may affect how such studies are framed, which participants are included, how data is collected and subsequently what findings are spotlighted. While women’s experiences of GDM healthcare are often broadly included in reviews, they are not often the exclusive focus with healthcare experiences folded into accounts of living with GDM [11], healthcare service implementation [12], diabetes and pregnancy [13], understanding of future risk [14] and seeking postpartum care after GDM [15].

 To address this gap, the aim of this review was to map the literature, identify gaps in knowledge and investigate the ways research has been conducted into GDM healthcare experiences. The research questions were:

1. When, where and how has knowledge been produced about women’s experiences of GDM healthcare?
2. What findings have been reported about women’s experience of GDM healthcare?

## Methods

A scoping review was selected as the most appropriate method given our multiple aims relate to mapping the field of GDM healthcare experiences [18]. The reporting of this scoping review was guided by an adaptation of the PRISMA-ScR (Preferred Reporting Items for Systematic reviews and Meta-Analyses extension for Scoping Reviews) reporting guidelines [19].

### Search strategy

The search strategy was designed in consultation with a research librarian. The following databases were used: Scopus, PubMed, CINAHL, Web of Science, MEDLINE, Embase and Joanna Briggs Institute EBP. These databases were searched on 27 September 2021 by the first author using the keywords and MESH terms outlined in Table 1. No limits were set on publication date, study design or country of origin. The reference lists of included articles were also examined to identify other potential articles (i.e. snowballing).

**Table 1:** Databases searched

Database	Keywords/MESH terms
CINAHL	((MM “Diabetes Mellitus, Gestational”) OR ((MH “Diabetes Mellitus+”) AND (MH “Pregnancy+”))) AND (“patient experience” OR (MM “Life experiences+”) OR (MH “Attitude to Health+”))
EMBASE, Joanna Briggs Institute EBP Database	(‘Gestational diabetes’ OR ‘Diabetes in pregnancy’ OR ‘pregnancy induced diabetes’ OR ‘pregnancy diabetes’ OR (diabetes mellitus AND pregnancy)) AND (‘women’s views’ OR ‘lived experience’ OR ‘women’s experience’ OR ‘patient experience’ OR ‘health knowledge, attitudes, practice OR attitudes to health’)
MEDLINE	(‘Gestational diabetes’ OR ‘Diabetes in pregnancy’ OR ‘pregnancy induced diabetes’ OR ‘pregnancy diabetes’ OR (diabetes mellitus AND pregnancy)) AND (‘women’s views’ OR ‘lived experience’ OR ‘women’s experience’ OR ‘patient experience’ OR ‘health knowledge, attitudes, practice OR attitudes to health’)
PubMed	(“gestational diabetes“[All Fields] OR “pregnancy diabetes mellitus“[All Fields] OR “pregnancy diabetes“[All Fields] OR “pregnancy induced diabetes“[All Fields] OR “diabetes in pregnancy“[All Fields]) AND (“women’s experiences“[All Fields] OR “life experiences“[All Fields] OR “health knowledge“[All Fields] OR “patient experience“[All Fields] OR “health attitude“[All Fields])
Scopus	((TITLE-ABS-KEY (“gestational diabetes”) AND ALL (experience*))) AND NOT INDEX (Medline)
Web of science	TOPIC:(“gestational diabetes” experience*)

### Study selection

References were downloaded into Endnote before being exported into the online systematic review platform Rayyan [20]. Titles and abstracts were first screened against inclusion criteria by the first author and uncertainties about article inclusion were referred to the second and third authors for a decision. A second reviewer independently screened a subset (5%) of titles and abstracts of studies for eligibility to ensure inclusion criteria were consistently applied. Studies were included if they reported primary (empirical) research in the English-language in a published peer-reviewed journal. Studies had to have an explicit aim of focusing on GDM and include direct reporting of participants’ experiences of healthcare. The experience of healthcare is here understood as being the patient experience of care occurring in formal clinical settings, including interactions with providers and other aspects of care prescribed by healthcare professionals. Exclusion criteria were reviews of any kind, research that was not empirical (e.g. personal accounts) and conference abstracts.

### Data extraction and synthesis

Data from studies including authors, year published, study design, setting, sample size, recruitment site, stated theoretical approach, data collection method, languages and findings, were extracted into a custom template developed in Microsoft Excel. Findings were further summarised through an iterative coding process and used to develop a series of categories that broadly captured women’s experiences of GDM healthcare.

## Results

### Search results

**Fig. 1:**
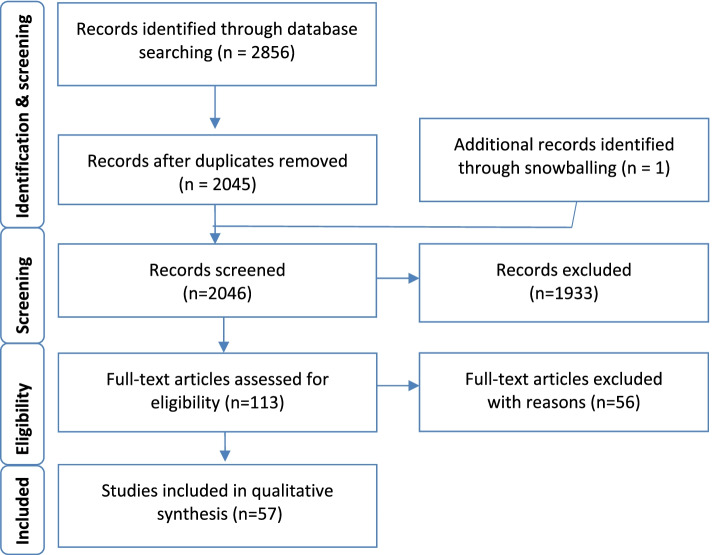
The process of data gathering

A total of 2856 articles were identified as potentially relevant to the research question from database searches. After removing duplicates (*n* = 811) and excluding non-relevant studies by screening titles and abstracts (*n* = 2045) and identifying an additional study through snowballing (*n* = 1), 112 articles were examined for inclusion through a full text assessment. Of these, 57 articles were included in this review, with 55 studies being excluded with reasons for exclusion documented. Figure 1 outlines the process of data gathering and Additional file: Appendix 1 for summarised study characteristics.

### Publication dates

**Fig. 2:**
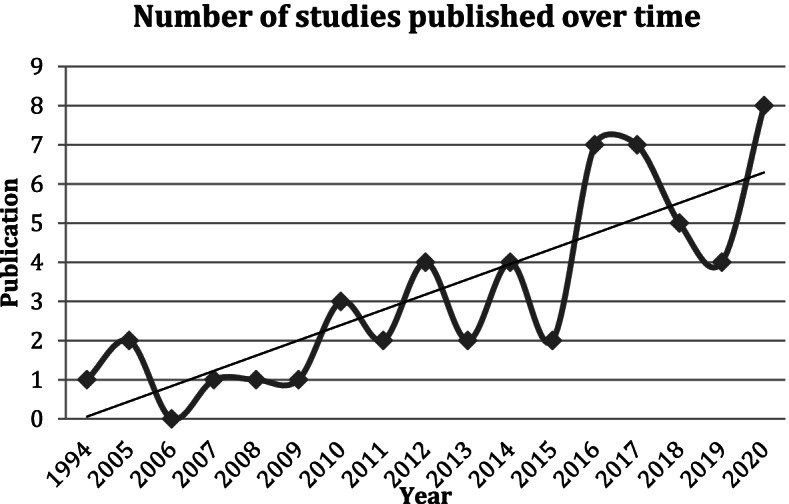
Included studies published over time

All of the included studies were published from 2005 onwards, except for one early study published in 1994 [21]. There has been an overall increase in the number of studies published each year to 2020 (see Fig. 2).

### Research settings

For the vast majority of studies (*n* = 55, 91%), recruitment of women with GDM was conducted via hospitals, clinics and healthcare providers, with one of these studies also conducting additional recruitment via workplaces [22]. Electronic databases were used in two studies for recruitment, with one study using a national diabetes database in Australia [23] and another using electronic health data in the United States [24]. Two studies which targeted Indigenous populations relied on pre-existing relationships; a Canadian study gained entry to an Indigenous population by building on pre-existing relationships with the Mi’kmaq communities [25] and an Australian study which focused on Aboriginal populations relied on existing research networks [26]. Only one study recruited completely outside clinical, healthcare and research settings using advertisements and community notices in targeted areas of Atlanta, Georgia in the United States [27].

**Fig. 3:**
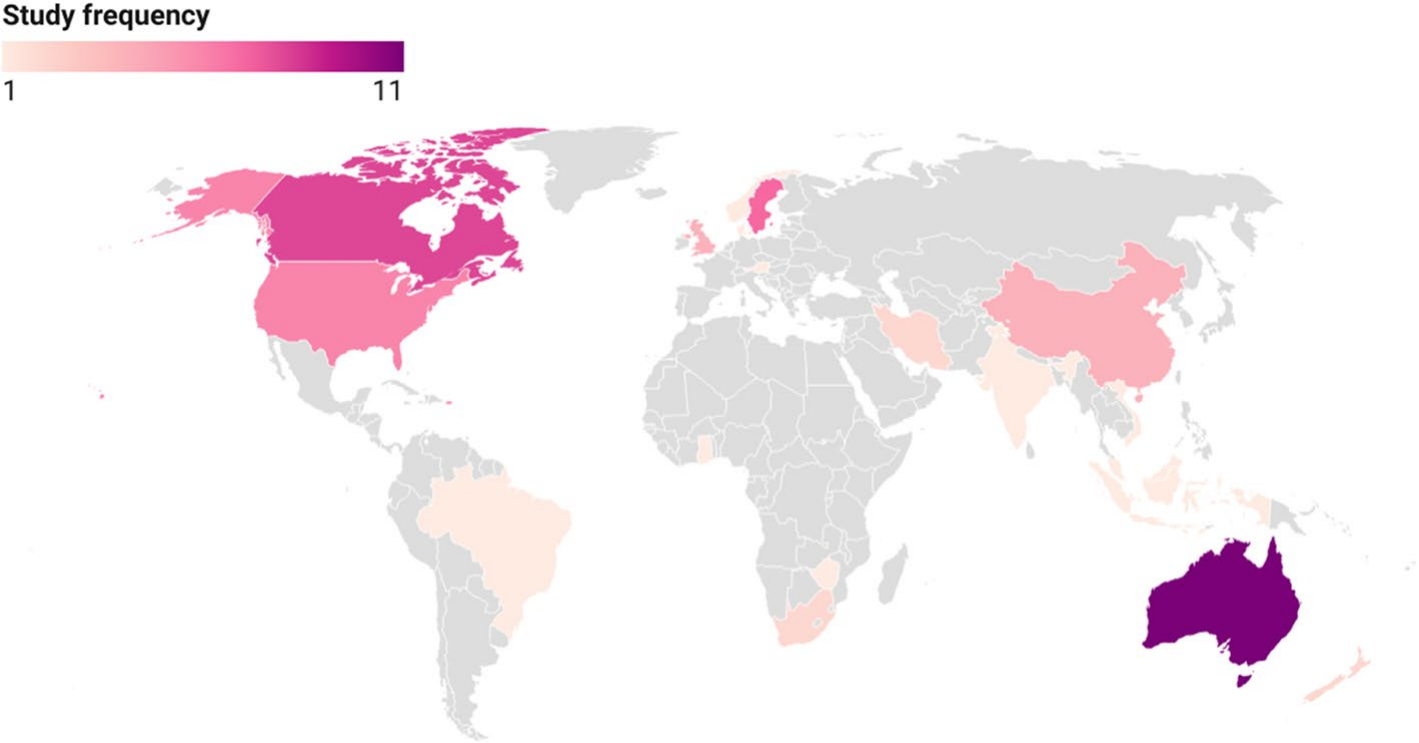
Settings of included studies

A handful of studies (*n* = 5, 9%) were based in countries classified as low- and lower middle-income; there were no countries considered ‘least developed’ [28]. For the most part, included studies were concentrated in a relatively small number of high-income countries, with the top six countries for research on women’s experiences of GDM healthcare being Australia (*n* = 11), Canada (*n* = 8), Sweden (*n* = 7), the United States (*n* = 6), the United Kingdom (*n* = 4) and China (*n* = 4). The remaining studies were spread across a number of countries, largely one study per setting: Austria [29], Brazil [30], Denmark [31], Ghana [32], India [33], Indonesia [34], Iran [35, 36], Malaysia [37], New Zealand [38, 39], Norway [40], Singapore [41], South Africa [42, 43], Vietnam [44], Zimbabwe [45] (see Fig. 3).

Forty-eight of the studies (84%) were conducted with participants in urban areas and the remaining studies (*n* = 9) were conducted in regional and rural areas of Australia [26, 46], Canada [25, 47–49], China [50], Tamil Nadu in India [33], and the state of New York in the United States [51]. A number of studies were conducted by the same research team and published in multiple installments; these studies were conducted in Lund, Sweden (6 studies), southeastern China (4 studies) and Melbourne, Australia (4 studies).

### Participants

The majority of studies specifically focused on women diagnosed with GDM as the sole target group, though two studies also interviewed comparative groups of women with different conditions such as DM [27, 52]. Several studies targeted women as well as healthcare professionals, including nurses, clinicians, general practitioners, with data being compared between groups [26, 27, 32, 36, 41, 46, 47, 53, 54]. In one study it was noted how some participants had pre-existing medical conditions, such as hypertension and HIV, and that their co-morbidities directly contributed to their perspective on GDM [36].

Depending on the nature of the study design—whether qualitative, mixed methods or quantitative—the range of participants varied greatly, from a small number of interview and focus group participants (*n* = 8) [55] through to large datasets such as the open-ended responses on a cross-sectional survey (*n* = 393) [23]. While there was some stratification of participants based on individual factors, such as body mass index [56] as well as glycaemic targets set [38], the main categorisation made was often in relation to ethnicity in studies from countries such as Australia, Sweden and the United States, where the focus on ethnic differences was built into the design of studies. For example, this included directly comparing ethnic groups, such as Swedish-born versus African-born [57], or comparing groups of women by their ethnicity, namely Caucasian, Arabic and Chinese [58].

### Study designs

The studies varied in how they understood, described and measured women’s experiences of GDM healthcare. Of the 57 included studies, 50 (88%) used qualitative study designs. Only four studies (7%) had quantitative designs and three (5%) employed mixed-methods [29]. The vast majority of studies (*n* = 49, 86%) were cross-sectional, with seven studies [21, 51, 56, 59–62] interviewing the same women at multiple time points. In terms of methodologies used, all the qualitative studies featured various types of interviews and/or focus groups. These were largely conducted face-to-face or via telephone. Seven studies employed more than one qualitative method to collect data [36, 43, 47, 55, 63–65] and, in addition, three studies used mixed methods to collect data [29, 41, 46]. One study focused on First Nations women in Canada used a focused ethnographic approach [49], and another 2021 study focused on South Asian women in Australia using ethnography [54]. The quantitative studies comprised four survey studies using questionnaires [37, 38, 52, 66].

### Theoretical approaches

The majority of studies did not specify a theoretical approach (*n* = 31, 54%), and relied on general data analysis approaches such as thematic analysis. Where a theory was referred to, it was largely used as a guiding framework for study design and data collection, and data analysis where applicable (see Additional file: Appendix 1). The three most popular theoretical approaches were the Health Belief Model (*n* = 6), Grounded Theory (*n* = 3) and phenomenology (*n* = 8), with the last of these specifically including hermeneutic [67] and interpretative approaches [63, 68]. Two of the studies that focused on Indigenous populations used culturally-sensitive qualitative methodologies designed to respect and recognise Indigenous worldviews, namely the Two-Eyed Seeing Approach [25] and the Kaupapa Māori methodology [39]. Another study [47] focused on an Indigenous population discussed qualitative research in general being the most “flexible and interpretive methodology” and how using open-ended interviewing creates a dialogue which recognises Indigenous oral traditions and knowledge.

### Data collection

Studies varied in when they captured data during the pregnancy and postpartum periods. Where the focus of a study was specifically on healthcare, women’s experiences were often elicited by researchers directly; otherwise, healthcare experience was generally revealed in relation to broader questions within the research framing, such as looking at factors that influence migrant women’s management of GDM [69, 70] or examining barriers and possible solutions to nonadherence to antidiabetic therapy [71].

Almost all studies were conducted in a primary language of the research team, with fluency in the primary language largely requisite for participation. However, there were 14 studies involving multicultural populations that allowed women to use their preferred language as research teams consisted of multilingual researchers, research assistants or interpreters (see Table 2).

**Table 2.** Languages used to collect data with multicultural study populations

**First author & year**	**Setting**	**Languages**
1. Hjelm, K. (2005) [72]	Sweden	Swedish, Arabic
2. Hjelm, K. (2007) [73]	Sweden	Swedish, Arabic
3. Bandyopadhyay, M. (2011) [62]	Australia	English, Bengali and Hindi
4. Hjelm, K (2012) [74]	Sweden	Swedish, Arabic
6. Jirojwong, S. (2017) [70]	Australia	Vietnamese, Thai, Laotian, Khmer
7. Razee H. (2010) [58]	Australia	English, Arabic, Mandarin, Cantonese
8 Carolan-Olah, M. (2017) [71]	United States	English, Spanish
10. Hjelm, K. (2018) [60]	Sweden	Swedish, other (interpreters/translators were used)
9. Dayyani I. (2019) [75]	Denmark	English, Danish, Arabic
11. Dickson, L.M. (2020) [42]	South Africa	English, other (interpreters/translators were used)
12. Muhwava, L.S. (2020) [43]	South Africa	English, Afrikaans, isiXhosa
13. Pace, R. (2020) [48]	Canada	English, Cree
14. Bandyopadhyay (2021) [54]	Australia	English, Hindi/Urdu

### Study findings on women with GDM experiences of healthcare

The findings from the 57 included studies were categorised into a number of salient aspects of formal healthcare experience, then further categorised as being positive and/or negative experiences depending on how participants’ self-reports were described and quoted by study authors. Where there was not an explicit reference to sentiment in the study, it has not been recorded in this review.

### Mental distress

Mental distress included acute emotional reactions such as shock and stress, as well as ongoing psychological challenges in coping with GDM. The vast majority of included studies noted mental distress of some kind (*n* = 48, 84%), inferring that mental distress was inextricably part of women’s experiences of GDM and intertwined with healthcare experience.

### Patient-provider interactions

From the moment diagnosis of GDM occurs, a cornerstone of women’s healthcare experience is interactions with providers, which differs depending on the model of care offered. ‘Interactions’ can be broadly defined as interpersonal encounters where communication occurs directly through conversations at consultations as well as group sessions, or interactions via other means such as text messages, emails and phone calls. Forty-four studies (*n* = 44, 77%) discussed patient-provider interactions in their findings; these were positive experiences (*n* = 9, 20%), negative experiences (*n* = 16, 36%), or ambivalent, being both positive and negative (*n* = 19, 43%). As an example of positive experience, one study reported “women were happy with the care provided in managing their GDM, acknowledging that the care was better than in their home country.” [62] In terms of negative experiences, women felt, for example, healthcare providers could be “preachy” [55] and discount their own expertise in their bodies [21]. One study [40] specifically examined the difference in women’s experiences with primary and secondary healthcare providers, and found that overall they received better care from the latter. More generally, the participants from one study emphasised the importance of a humanistic approach to care [76].

### Treatment satisfaction

Treatment satisfaction was a measure reported in two quantitative studies [37, 52], and the mixed-methods study [29]. The Diabetes Treatment Satisfaction Questionnaire (DTSQ) was used in two studies to measure satisfaction [29, 37]. The study by Anderberg et al. [52] used its own purposely developed instrument and found 89% of women with GDM marked “satisfied”, 2% marked “neutral” and no one indicated dissatisfaction. In the study by Hussain et al. [37], which used the DTSQ, 122 (73.5%) patients reported they were satisfied with treatment and 44 (26.5%) were unsatisfied; overall, the majority of patients were satisfied with treatment but retained a ‘negative’ attitude towards GDM. The study by Trutnovsky et al. [29] went further in its analysis as women responded to the DTSQ at three different phases – before treatment, during early treatment and during late treatment – and found that overall treatment satisfaction was high, and significantly increased between early and late treatment.

### Diet prescribed

Diet is a fundamental component of treatment for GDM. Once diagnosed, many women are prescribed modified diets to maintain blood sugar levels, which they record on paper or by using an electronic monitor at specified times. Thirty-nine studies (*n* = 39, 68%) included findings and discussion about women’s experiences of prescribed diet, and of those studies (*n* = 33, 84%) this is captured as generally a negative experience. In some studies, women’s experience of the prescribed diet was reported as being both positive and negative (*n* = 4, 10%); only one study (*n* = 1, 3%) recorded it as a positive experience [38]. The difficulty of following a new diet during pregnancy was a key reason as to why the experience was negative, as well as practical considerations such as being able to easily access fresh food in remote areas [26]. In studies with multicultural populations, negative experience related to managing the advice in conjunction with culturally-based diets. As noted in the two studies led by Bandyopadhyay, women had difficulty maintaining their traditional diet due to the new restrictions placed upon them [54, 62].

### Medication prescribed

Medication for GDM primarily involves some form of insulin, which is prescribed to manage blood sugar levels. Twenty-one studies (*n* = 21, 37%) included findings and discussion about women’s experiences of GDM medication and of those, it was mostly reported as being a negative experience (*n* = 13, 62%), with various reasons captured including insufficient time to “figure things out” [77] and causing feelings of anxiety and failure [78]. However, in a few studies prescribed medication was noted as being a positive experience (*n* = 3, 14%), or both a positive and negative experience (*n* = 5, 24%). In one study, a participant stated, “the fact that I’m on insulin makes it easy” [68].

### Monitoring

Monitoring captures both the direct monitoring conducted by healthcare providers, primarily blood and blood sugar level tests as well as ultrasounds, as well as self-monitoring women were required to carry out and which was often then verified by healthcare professionals. Twenty studies (*n* = 20, 35%) included findings and discussion about women’s experiences of monitoring and of those it was seen as being negative (*n* = 14, *n* = 70%), both positive and negative (*n* = 5, 25%) and positive (*n* = 1, *n* = 5%). In the one study that reported positive experiences only, a participant reported that she thought it was good “they are monitoring us all the time” [30]. Studies reporting negative experiences with monitoring had participants citing reasons such as feeling over-scrutinised [65].

### Access to timely healthcare

Access to healthcare can be a challenge in certain settings, and, even when access is possible, timeliness can be an issue. Of the 31 studies (*n* = 31, 54%) that referred to access in their findings, the vast majority of these studies (*n* = 28) reported access to timely healthcare being a negative experience, with reasons cited including geographic distance [39, 46], difficulties in being able to make a booking to be seen at a hospital [79] and then, when being seen, not having enough time with a healthcare provider [27, 44]. In one of the two studies reporting positive experiences [52], all questions relating to accessibility indicated satisfaction (97%); in the other of the two studies [38], the majority of women (68%) appreciated that health professionals took time to listen and explain.

### Provision of information

Information to support women is critical in managing their GDM diagnosis. Ongoing management came from meetings with healthcare providers—described in one study as being “frontline support” [79]— alongside sources focused on diet, medication, exercise and other pertinent information. Across all the studies which discussed how provision of information by healthcare providers was received (*n* = 38, 67%), it was noted as largely negative (*n* = 24, 63%) and both positive and negative (*n* = 10, 18%), though there were discussions of positive experiences (*n* = 4, 7%). Considered together, all the studies suggested how crucial clear information is to a positive experience of healthcare. For women, having inadequate knowledge about how to cope was a source of disempowerment and, across the majority of studies (*n* = 44, 77%), participants reported they found information from providers was insufficient. Interestingly, one of these studies found the insufficiency was actually due to the information being “too much” [26], while another study [59] found there was a desire for “more frequent controls and dietary advice”. The inappropriate timing of information was also reported in a number of studies [31, 58, 79–81]. One study noted how participants found one group of healthcare providers, midwives and nurses provided better information than general practitioners [40], while another noted the contradictory nature of advice from different providers [82]. Language barriers were also identified as a problem with information provision with a lack of information available in a woman’s preferred language [69].

### Financial issues

Direct healthcare costs including out-of-pocket medical consultation fees, medication and medical equipment were primarily raised by participants in the United States [27], Ghana [32] and Zimbabwe [45], with the last of these reporting that some participants discussed “the related costs of treatment … resulted in participants foregoing some of the tests and treatments ordered” [45]. A study from Canada noted a number of participants with refugee status discussed the “economic challenge” of managing GDM and that the cost of diabetes care “was quite high and difficult to manage” [83]. Several indirect costs were also discussed across the studies. In a number of studies (*n* = 7), the additional cost of purchasing healthy food to manage GDM was brought up as being a burden [25, 27, 38, 42, 48, 51, 84]. However, in one study, women said the costs related to food went down as being able to buy take-away (fast foods) became restricted [38]. Loss of income [46] as well as daycare costs were cited [25], as was additional transportation and hospital parking costs [39, 46, 56]. Finally, women in one study reported having to change occupations and even quit work to manage GDM [21].

## Discussion

The growing number of research studies relaying women’s GDM healthcare experience is encouraging, given increasing incidence and health burden. As this review demonstrates, there are important commonalities across all studies, suggesting that some aspects of GDM healthcare experience seem to be universal; mental distress, for example, was reported in most studies. In contrast, other aspects of GDM healthcare experience seem to relate to factors specific to local settings; financial issues were mainly raised in settings where healthcare is not universal or is not readily affordable. Related financial issues were raised by participants in a number of rural-based studies, revealing something of a difference between urban and rural healthcare settings regardless of country context.

All of the included studies relied on women’s self-reporting without necessarily involving other measures, which broadly fell into two categories: women currently undergoing care for GDM at the time of study data collection and those looking back on past experience. Included studies were overwhelmingly qualitative in design, with relatively small numbers of participants for each category; put together, though, they paint a broad picture of women’s GDM healthcare experience across a range of settings. As the phenomenon being examined here is women’s experiences, qualitative methodologies are vital given the experience of health, illness and medical intervention cannot be quantified [85]. On the other hand, quantitative studies are able to include far more participants, though it is important to note not necessarily greater applicability and generalisability; when both types of studies are considered together as in mixed-methods study designs, there is a possibility of corroboration, elaboration, complementarity and even contradiction [85].

Recruiting women through clinical and other healthcare settings, as almost all of the included studies did, necessarily leads to biased samples of participants likely to be ‘compliant’ with healthcare requirements and treatment regimens. As one study noted, compliance was high despite limited understanding of GDM and dietary requirements, as well as why change was required [71]. This scenario occurs against the backdrop of the inherent power imbalance which exists in patient-provider relationships in reproductive healthcare [86]. A few of the included studies demonstrated reflexivity for this issue, with the studies most sensitive to these concerns focused on Indigenous populations. This power imbalance also exists in patient-researcher relationships [87]; a critical way to mitigate this effect is to actively include participants in research design, which only one included study reported doing 75]. This suggests an important direction for future studies, building on recent work involving patients to establish research priorities for GDM [88]. Indeed, many of the included studies did incorporate ideas about improving healthcare as proposed by the women themselves. For example, in one study, participants reported that small group sessions with medical practitioners and more detailed leaflets would be useful [44], suggesting how current sessions could be run better.

Culturally sensitive qualitative methodologies were employed with Indigenous populations and those learnings could be further extended to other groups of research participants. GDM is known to be more common in foreign-born racial minorities [9], so it is encouraging that some studies focused on these particular groups and had study designs that included interpreters. However, this line of research is arguably under-developed given most studies excluded minoritised women who did not have a high degree of fluency in the dominant language. Language barriers were identified as a problem with information provision with GDM healthcare [69, 70], and it is possible to extend this idea to research contexts themselves. Not being able to use the language one feels most fluent in clearly affects the way GDM healthcare experiences are reported.

Treatment satisfaction was used in both quantitative and mixed-method studies, but as a solo measure the insights it can provide is limited; we do not exactly know why or how, for example, women’s satisfaction improves later in GDM care [29]. However, a number of the studies provide possible answers. Persson et al. [61] describe the process women underwent “from stun to gradual balance” due to a process of adaptation that became easier “with increasing knowledge” about how to self-manage GDM. Ge et al. [89] found that women developed a philosophical attitude over time to reach a state of acceptance, and such a shift in attitude would clearly have an impact on how healthcare is received and understood. These findings suggest the benefit of both time and experience, and the role of these factors could be better examined with more longitudinal studies.

In this scoping review, under half of the included studies explicitly drew on theory. But as argued by Mitchell and Cody [90], regardless of whether it is acknowledged, theoretical interpretation occurs in qualitative research. Explicitly incorporating theoretical approaches are valuable in strengthening research design when such conceptual thinking clearly informs the research process; here, examining women’s lived experiences without articulating the theoretical bases which underpins research design and analysis leads to a lack of acknowledgement of relevant context as to how both treatment and research occurs. For example, gender exerts a significant influence upon help-seeking and healthcare delivery [91], and particularly for GDM. In future, it might be useful to further consider the value of theory in elucidating women’s experiences to address biases in research design to further the fields of study which relate to women’s GDM experiences [90].

Finally, much of this research has been generated in a small number of wealthy countries. GDM is a growing problem in low income settings and yet, as Nielsen et al. [92] describe, detection and treatment of GDM is hindered due to “barriers within the health system and society”. Going further, Goldenberg et al. suggest that due to competing concerns, “diagnosing and providing care to women with diabetes in pregnancy is not high on the priority lists in many LMIC”. [93] Similar barriers exist with GDM research endeavours; ensuring that evaluation of healthcare includes women’s experiences of GDM healthcare would be valuable to researchers in these settings and beyond. Thus there are clear gaps in practice as well as the research literature in considering women’s experiences of GDM healthcare internationally.

### Implications

Research into women’s experience of GDM healthcare continues to accumulate and continued research efforts will contribute to far greater understanding of how we might best support women and improve healthcare outcomes. However, there is room for improvement, such as by following participants longitudinally, using mixed methods and taking more reflexive and theoretically informed approaches to researching women’s experiences of GDM healthcare. There is a need highlighted for more culturally sensitive research techniques as well as including women in the study design process, and not just as research subjects to be instrumentalised for developing recommendations for clinical delivery.

### Strengths and limitations

Secondary analyses of primary research are challenging to conduct when the pool of included studies is highly heterogeneous. In this scoping review, in order to synthesise a large group of diverse studies, summarising results in terms of positive and negative experiences of GDM healthcare was reductive but necessary. This key strength of our review, inspired by sentiment analysis [94], shows the utility in capturing overall polarity of feelings as it highlights the ambivalence of healthcare experience. An additional strength was involving a research librarian to help design the searches and advise on relevant databases.

There are several limitations. For our search strategy, we used a broad set of terms relating to patient experience, but there is no standard set of terminology about this type of research, so it is possible some studies were missed. Only studies in English were included, so any studies published in other languages were missed. We did not conduct a critical appraisal on the included studies, which was a limitation; however, this was a purposeful choice in order to include a wide range of studies, including from research settings that are not as well-resourced.

## Conclusions

 This scoping review identifies commonalities in how GDM healthcare is delivered and received in settings around the world, with women’s experiences varying depending on what model of care is applied alongside other factors. Documenting experiences of GDM healthcare is a vital way to inform future policy and research directions, such as more theoretically informed longitudinal and mixed method approaches, and co-designed studies.

## Supplementary Information

**Additional file 1.** Characteristics of the studies included in the scoping review

## Data Availability

All data generated or analysed during this study are included in this published article and its supplementary information files.
